# Antitumor activity of colloidal silver on MCF-7 human breast cancer cells

**DOI:** 10.1186/1756-9966-29-148

**Published:** 2010-11-16

**Authors:** Moisés A Franco-Molina, Edgar Mendoza-Gamboa, Crystel A Sierra-Rivera, Ricardo A Gómez-Flores, Pablo Zapata-Benavides, Paloma Castillo-Tello, Juan Manuel Alcocer-González, Diana F Miranda-Hernández, Reyes S Tamez-Guerra, Cristina Rodríguez-Padilla

**Affiliations:** 1 Laboratorio de Inmunología y Virología. Departamento de Microbiología e Inmunología. Facultad de Ciencias Biológicas de la Universidad Autónoma de Nuevo León, San Nicolás de los Garza, N. L. México

## Abstract

**Background:**

Colloidal silver has been used as an antimicrobial and disinfectant agent. However, there is scarce information on its antitumor potential. The aim of this study was to determine if colloidal silver had cytotoxic effects on MCF-7 breast cancer cells and its mechanism of cell death.

**Methods:**

MCF-7 breast cancer cells were treated with colloidal silver (ranged from 1.75 to 17.5 ng/mL) for 5 h at 37°C and 5% CO_2 _atmosphere. Cell Viability was evaluated by trypan blue exclusion method and the mechanism of cell death through detection of mono-oligonucleosomes using an ELISA kit and TUNEL assay. The production of NO, LDH, and Gpx, SOD, CAT, and Total antioxidant activities were evaluated by colorimetric assays.

**Results:**

Colloidal silver had dose-dependent cytotoxic effect in MCF-7 breast cancer cells through induction of apoptosis, shown an LD_50 _(3.5 ng/mL) and LD_100 _(14 ng/mL) (*P < 0.05), significantly decreased LDH (*P < 0.05) and significantly increased SOD (*P < 0.05) activities. However, the NO production, and Gpx, CAT, and Total antioxidant activities were not affected in MCF-7 breast cancer cells. PBMC were not altered by colloidal silver.

**Conclusions:**

The present results showed that colloidal silver might be a potential alternative agent for human breast cancer therapy.

## Background

Prior to 1938, colloidal silver was widely used to prevent or treat numerous diseases. Its use decreased with the development of antibiotics, such as penicillin and sulfanilamide [1]. However, since 1990 there has been a resurgence on the use of colloidal silver as an alternative medicine because of increased resistance of bacteria to antibiotics, and the continuing search for novel and affordable antimicrobial agents. Colloidal silver is a suspension of submicroscopic metallic silver particles of about 0.001 microns in size, the presence of particles results in the overall increased surface area [2,3]. Colloidal silver has been used as disinfectant of foods and water in Mexico; it acts by disabling the oxygen metabolism enzymes in bacteria, which ultimately kills microorganisms. *In vitro *evidence has shown that bacterial isolates of *Escherichia coli *and *Staphylococcus aureus *are highly susceptible to colloidal silver treatment [4].

Although the use of colloidal silver as an antimicrobial agent is recognized [4], there are scarce reports on its use as antitumor agent; among these, there is a recent report on the anti-proliferative effect of silver nanoparticles on human glioblastoma cells (U251) *in vitro *[5]. Cancer is an important cause of mortality worldwide and the number of people who are affected is increasing, being the breast cancer one of the major causes of death in women [6]. The origin of cancer cells can be related to metabolic alteration, such as mitochondrial increase of glycolysis, which largely depends on this metabolic pathway needed to convert glucose into pyruvate, for the generation of ATP to meet cancer cell energy needs. Many cancer cell types produce ATP by conversion of glucose to lactate and exhibit lower oxidative phosphorylation, and accelerated glycolysis ensures ATP levels compatible with the demands of fast proliferating tumor cells in a hypoxic environment [7,8]. Furthermore, many reports have shown cellular changes resulting from oxidative stress produced by the generation of reactive oxygen intermediates (ROI) in tumor cells, which increases the cytotoxicity activity of the drugs [9]; the oxidative stress is a loss of balance between ROI production and intracellular antioxidants such as superoxide dismutase (SOD), catalase (CAT), glutathione peroxidase (Gpx), and extracellular antioxidants.

Although there is a wide range of cytotoxic agents used in the treatment of breast cancer, such as doxorubicin, cisplatin, and bleomycin, they have shown drawbacks in their use and are not as efficient as expected [10]. Therefore, it is of great interest to find novel therapeutic agents against cancer. Hence, we evaluated the effects of colloidal silver on MCF-7 human breast cancer cells growth.

## Methods

### Main reagents

Penicillin-streptomycin solution, ficoll-hypaque solution, trypsin-EDTA solution, RPMI-1640 medium, Dulbecco's modified Eagle's medium (DMEM/F-12), and 1% antibiotic-antimycotic solution were obtained from (Life Technologies GIBCO, Grand Island, NY, USA). Fetal bovine serum (FBS) was purchased from Sigma-Aldrich (St. Louis, MO).

### Cell Culture

MCF-7 human breast cancer cell line was purchased from American Type Culture Collection (ATCC, Manassas, VA, USA) and was maintained in Dulbecco's modified Eagle's medium supplemented with 10% fetal bovine serum (FBS) and 1% antibiotic-antimycotic solution. Cells were grown to confluence at 37°C, and 5% CO_2 _atmosphere.

### Isolation of peripheral blood mononuclear cells (PBMC)

Blood from healthy human volunteers was obtained with heparinized syringes and was placed into sterile polypropylene tubes. PBMC were further isolated by hystopaque 1077 density gradient centrifugation at 400 g for 30 min at 25°C (Sigma-Aldrich, St. Louis MO, USA). PBMC were then washed twice with FBS-free medium (RPMI-1640) at 250 g for 10 min at 25°C and adjusted to 5 × 10^3 ^cells/well for analysis.

### Colloidal silver

The grenetine-stabilized colloidal silver was purchased from MICRODYN (Mexico, D.F.) as a 0.35% stock solution. It was filtered and diluted to a concentration of 1.75 ng/mL with DMEM/F-12 or RPMI-1640 medium.

### Cell viability

Cells (5 × 10^3 ^cells/well) were plated on 96 flat-bottom well plates, and incubated 24 h at 37°C in 5% CO_2 _atmosphere. After incubation, culture medium was removed, and colloidal silver diluted in the same medium was added at concentrations ranging from 1.75 to 17.5 ng/mL. The plates were then incubated for 5 h at 37°C, and 5% CO_2 _atmosphere. Thereafter, the supernatant was removed and cells were washed twice with DMEM/F-12 medium. Cell viability was determined by the trypan blue exclusion method, and cytotoxicity was expressed as the concentration of 50% (LD_50_) and 100% (LD_100_) cell growth inhibition. Results were given as the mean + SD of three independent experiments.

### Mechanism of cell death analysis

Cell death type was assessed by the detection of mono-oligonucleosomes (histone-associated DNA fragments) using an ELISA kit (Cell Death Detection ELISA PLUS, Roche Applied Science, IN, USA) following the manufacturer's instructions. In brief, the cytoplasmic lysates from untreated controls and colloidal silver treated cultures were transferred to a streptavidin-coated plate supplied by the manufacturer. A mixture of anti-histone biotin and anti DNA-POD were added to cell lysates and incubated for 2 h. The complex was conjugated and then the plate was read at a wavelength of 405 nm. The increase in mono-oligonucleosomes production in cells lysates was calculated as the ratio of the absorbance of colloidal silver treated cells/absorbance of untreated control. Results were given as the mean + SD of three independent experiments.

### Tunel

Terminal deoxynucleotidyl transferase-mediated dUTP nick end-labeling (TUNEL) was performed with TACS 2 TdT-DAB In Situ Apoptosis Detection kit (Trevigen, Gaithersburg, Maryland, USA), following the manufacturer's instructions. Briefly, after culture MCF-7 cells at 10^6 ^cells/well and treated with LD_50 _and LD_100_, by 5 h, the cells were digested with proteinase K at a concentration of 20 μg/mL for 15 minutes. Endogenous peroxidase activity was quenched with 2% H_2_O_2 _for 5 minutes. The cells were immersed in terminal deoxynucleotidyl transferase (TdT) buffer. TdT, 1 mM Mn^2+^, and biotinylated dNTP in TdT buffer were then added to cover the cells and incubated in a humid atmosphere at 37°C for 60 minutes. The cells were washed with PBS and incubated with streptavidin-horseradish peroxidase for 10 minutes. After rinsing with PBS, the cells were immersed in DAB solution. The cells were counterstained for 3 minutes with 1% methyl green. Cells containing fragmented nuclear chromatin characteristic of apoptosis will exhibit brown nuclear staining that may be very dark after labeling.

### Detection of lactate dehydrogenase (LDH) activity

The conversion of lactate to pyruvate was detected using the Cytotoxicity Detection Lactate Dehydrogenase kit (Roche Applied Science, IN, USA) following the manufacturer's instructions. MCF-7 breast cancer cells and PBMC treated with colloidal silver were washed twice with ice-cold PBS, harvested by centrifugation at 250 g for 10 min at 25°C, and the supernatant was used for the activity assay according to the manufacturer's instructions. Optical densities resulting from LDH activity were measured in a microplate reader at 490 nm. Results were given as the mean + SD of three independent experiments.

### Nitrite determination

Accumulation of nitrite in the supernatants of control and treated MCF-7 and PBMC cultures was used as an indicator of nitric oxide production. Cells were incubated for 5 h in DMEM/F-12 medium, in the presence or absence of colloidal silver in triplicates, in a total volume of 200 μL DMEM/F-12 medium. After incubation, supernatants were obtained and nitrite levels were determined with the Griess reagent, using NaNO_2 _as standard. Optical densities at 540 nm were then determined in a microplate reader (Bio-Tek Instruments, Inc.).

### Determination of intracellular antioxidants

The antioxidants production was measured using the following kits: Cellular glutathione peroxidase (Gpx) assay kit (Oxford Biomedical Research, MI, USA), superoxide dismutase (SOD) assay kit (Cayman Chemical Company, MI, USA), and catalase (CAT) assay kit (Cayman Chemical Company, MI, USA) according to the manufacturer's instructions. Briefly, to determine the activity of Gpx, SOD, and CAT; MCF-7 and PBMC were incubated with LD_50 _(3.5 ng/mL) and LD_100 _(14 ng/mL) of colloidal silver for 5 h. Cells were then washed three times with PBS and sonicated on ice in a bath-type ultrasonicador (80 Watts output power) for 15-s periods for a total of 4 min; the solution was then centrifuged at 1500 g for 5 min at 4°C. The obtained supernatants were used to determine intracellular antioxidants in a microplate reader at 540 nm.

### Total antioxidant (extracellular antioxidants)

The total antioxidant production was determined using the Total Antioxidant Colorimetric Assay Kit (US Biological, Massachussets, USA) following manufacturer's instructions. Briefly, MCF-7 and PBMC were treated with LD_50 _(3.5 ng/mL) and LD_100 _(14 ng/mL) of colloidal silver for 5 h. Thereafter, supernatants were used to determine antioxidants in a microplate reader at 490 nm.

### Statistical analysis

Data represent the mean + SD of triplicates from three independent experiments. Statistical differences were obtained using the analysis of variance, and the Dunnett's and Turkey's tests (SPSS v. 12 program).

## Results

### Cytotoxic activity of colloidal silver on MCF-7 human breast cancer cells

**Figure 1:**
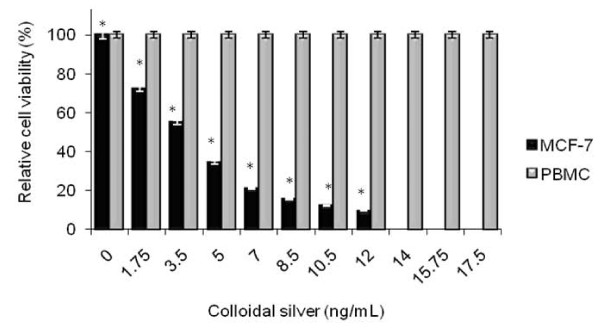
**Cell viability of MCF-7 cell line and PBMC treated with colloidal silver**. Cells (5 × 10^3 ^cells/well) were plated on 96 flat-bottom well plates, and incubated 24 h at 37°C in 5% CO_2 _atmosphere. After incubation, culture medium was removed, and colloidal silver diluted in the same medium was added at concentrations ranging from 1.75 to 17.5 ng/mL. The plates were then incubated for 5 h at 37°C, and 5% CO_2 _atmosphere. Thereafter, the supernatant was removed and cells were washed twice with DMEM/F-12 medium. Cell viability was determined by the trypan blue exclusion method, and cytotoxicity was expressed as the concentration of 50% (LD_50_) and 100% (LD_100_) cell growth inhibition. The experiments were performed in triplicates; data shown represent mean + SD of three independent experiments. *P < 0.05 as compared with untreated cells.

As observed in Figure 1, colloidal silver induced dose-dependent cytotoxic effect on MCF-7 breast cancer cells; the median lethal dose was (LD_50_) 3.5 ng/mL and the lethal dose (LD_100_) was 14 ng/mL (*P < 0.05). In contrast, colloidal silver treatment did not affect PBMC viability (Figure 1). These LD_50 _and LD_100 _were used in further experiments.

### Colloidal silver induced apoptosis in MCF-7 breast cancer cells

**Figure 2:**
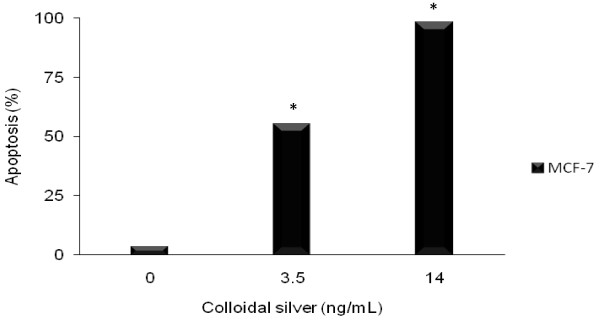
**Apoptosis mediated by colloidal silver on MCF-7 cell line**. MCF-7 cells were treated with increasing concentrations of colloidal silver (1.75 to 17.5 ng/mL) for 5 h. Thereafter, the levels of mono-oligo nucleosome fragments were quantified using the Cell Death Detection Kit. The experiments were performed in triplicates; data shown represent mean + SD of three independent experiments. *P < 0.05 as compared with untreated cells.

**Figure 3:**
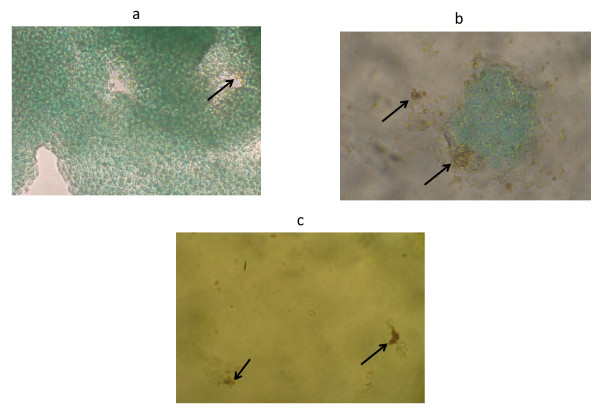
**MCF-7 cells stained by the TUNEL technique, counterstained with methyl green**. (**a**) MCF-7 control, showing few brown staining of cells (arrows). (**b**) MCF-7 treated with colloidal silver LD_50 _(**c**) and LD_100 _showing abundant brown staining of cells (arrows). Original magnifications, **a**, **b, and c**: 40 ×.

The colloidal silver induced the mechanism of cell death through apoptosis in MCF-7 human breast cancer cell line, determined by the detection of mono-oligonucleosomes. The effects of LD_50 _and LD_100 _in control cells only caused non-significant cytotoxicity of 3.05% (P < 0.05), respectively (Figure 2). The TUNEL technique was also used to detect apoptosis. Labeling of DNA strand breaks *in situ *by TUNEL demonstrated positive cells that were localized in MCF-7 cells treated with LD_50 _and LD_100 _and control, with increased cell apoptosis in the LD_50 _and LD_100 _(Figure 3).

### Effect of colloidal silver on the activity of lactate dehydrogenase in MCF-7 and PBMC

**Figure 4:**
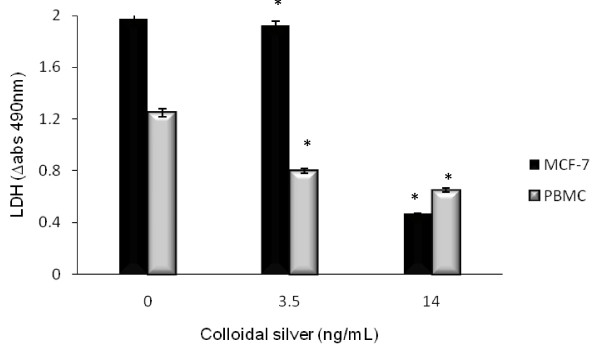
**Effect of colloidal silver on LDH activity in MCF-7 cells and PBMC**. LDH activity was measured by changes in optical densities due to NAD^+^reduction which were monitored at 490 nm, as described in the text, using the Cytotoxicity Detection Lactate Dehydrogenase kit. The experiments were performed in triplicates; data shown represent mean + SD of three independent experiments. *P < 0.05 as compared with untreated cells.

The lactate dehydrogenase activity significantly (*P < 0.05) decreased in MCF-7 and PBMC treated with colloidal silver LD_50 _and LD_100 _concentrations. Colloidal silver-treated MCF-7 LD_50 _and LD_100 _were 1.918 U/mL and 0.464 U/mL, respectively; untreated MCF-7 cells value was 1.966 U/mL. Similarly, colloidal silver-treated PBMC LD_50 _and LD_100 _concentrations were 0.964 U/mL and 0.796 U/mL, respectively; compared with the untreated PBMC value of 1.025 U/mL (Figure 4).

### Effect of colloidal silver on nitric oxide production in MCF-7 and PBMC

**Figure 5:**
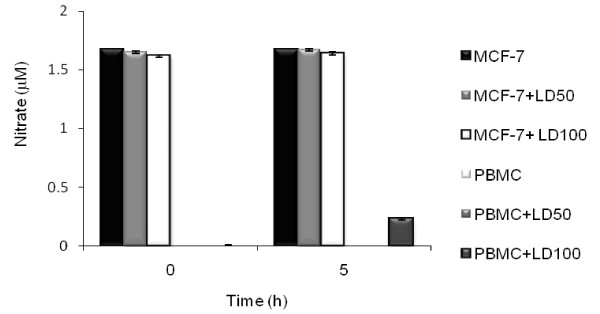
**Nitric oxide production in colloidal silver-treated MCF-7 and PBMC**. Nitric oxide production at 5 h by colloidal silver-treated MCF-7 and PBMC, was measured using the nitric oxide colorimetric assay kit, as described in methods. The experiments were performed in triplicates; data shown represent mean + SD of three independent experiments. *P < 0.05 as compared with untreated cells.

Figure 5 shows that NO production was undetectable (*P < 0.05) in untreated PBMC, and in colloidal silver-treated PBMC at LD_50 _and LD_100 _concentrations. However, in untreated MCF-7 cells, nitrites concentration was 1.67 μM, but the colloidal silver-treated MCF-7 at LD_50 _and LD_100 _did not affect NO production (*P < 0.05).

### Effect of colloidal silver on intracellular and extracellular antioxidants in MCF-7 and PBMC

**Figure 6:**
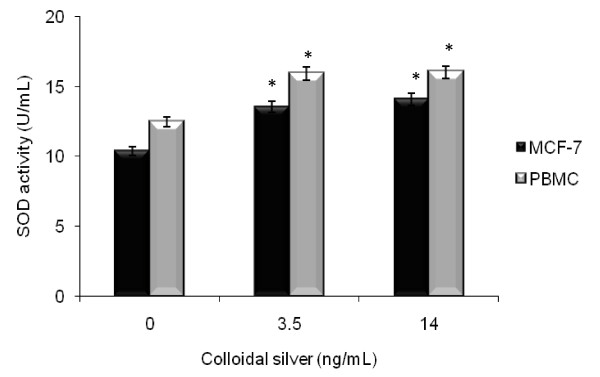
**Superoxide dismutase activity in colloidal silver-treated MCF-7 and PBMC**. MCF-7 breast cancer cells and PBMC were treated with colloidal silver for 5 h and then evaluated for superoxide dismutase (SOD) activity, as explained in methods. The experiments were performed in triplicates; data shown represent mean + SD of three independent experiments. *P < 0.05 as compared with untreated cells.

**Figure 7:**
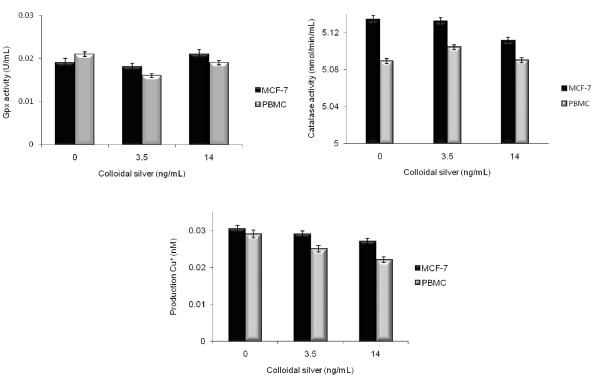
**Effect of the colloidal silver on the intracellular and extracellular antioxidants**. MCF-7 breast cancer cells and PBMC were treated with colloidal silver for 5 h and the antioxidants production were measured as described in methods, using colorimetric assay kits. **a) **Gpx activity, **b) **Catalase activity, **c) **Total antioxidant production. The experiments were performed in triplicates; data shown represent mean + SD of three independent experiments. *P < 0.05 as compared with untreated cells.

The superoxide dismutase activity was significantly (*P < 0.05) increased in colloidal silver-treated MCF-7 at LD_50 _(13.54 U/mL) and LD_100 _(14.07 U/mL) concentrations, compared with untreated control cells (10.37 U/mL), which also significantly (*P < 0.05) increased in colloidal silver-treated PBMC at LD_50 _(15.92 U/mL) and LD_100 _(16.032 U/mL) concentrations, compared with untreated PBMC (12.458 U/mL) (Figure 6). However, the catalase, glutathione peroxidase, and total antioxidant activities in MCF-7 and PBMC treated with colloidal silver did not differ significantly (*P < 0.05) from those of controls (Figure 7).

## Discussion

Woman breast cancer is the most important cause of mortality in the world [6]. Nowadays, some cytotoxic agents are used for its treatment including doxorubicin, daunorubicin, bleomycin, and cisplatin. However, they are costly and known to induce several side effects such as myelosuppression, anemia, and most importantly the generation of cellular resistance. For this, it is important to find alternative therapies or drugs to overcome these drawbacks [10]. Our *in vitro *studies showed that colloidal silver induced a dose-dependent cell death in MCF-7 breast cancer cell line through apoptosis, without affecting the viability of normal PBMC control cells. Most studies are focused on the effect of colloidal silver on bacterial growth, and the present study might contribute to the comprehension of this compound on cancer therapy. It has been known that cancer cells increased the rate of glycolysis; in this metabolic pathway lactate dehydrogenase is involved in catalyzing the conversion of pyruvate into lactate, which consumes NADH and regenerates NAD^+ ^[8]. In the present study, we showed that MCF-7 breast cancer cells treated with colloidal silver, significantly reduced the dehydrogenase activity, resulting in decreased NADH/NAD^+^, which in turn induces cell death due to decreased mitochondrial membrane potential. Death cell can also be produced by ROI (Reactive Oxygen Intermediates), and RNI (Reactive Nitrogen Intermediate) metabolites. Our results demonstrated that nitric oxide production was not affected by colloidal silver treatments, as compared with untreated cells (*P < 0.05), suggesting that the MCF-7 breast cancer cell death was independent of nitric oxide production. In addition, it was observed that colloidal silver did not affect the catalase and glutathione peroxidase activities (*P < 0.05). However, the colloidal silver treatment increased superoxide dismutase activity compared with untreated MCF-7 and PBMC (*P < 0.05). This may cause a redox imbalance, significantly increasing the SOD activity in response to the production of high levels of ROI molecules and the lack of activity of catalase and glutathione peroxidase may allow the toxic effect of hydrogen peroxide (H_2_O_2_) leading to cell death [10]. The H_2_O_2 _causes cancer cells to undergo apoptosis, pyknosis, and necrosis. In contrast, normal cells are considerably less vulnerable to H_2_O_2_. The reason for the increased sensitivity of tumor cells to H_2_O_2 _is not clear but may be due to lower antioxidant defenses. In fact, a lower capacity to destroy H_2_O_2 _e.g., by catalase, peroxiredoxins, and GSH peroxidases may cause tumor cells to grow and proliferate more rapidly than normal cells in response to low concentrations of H_2_O_2_. It is well known that H_2_O_2 _exerts dose-dependent effects on cell function, from growth stimulation at very low concentrations to growth arrest, apoptosis, and eventually necrosis as H_2_O_2 _concentrations increase [8]. This dose dependency may be shifted to the left in tumor cells, making them more sensitive to both the growth stimulatory and cytotoxic effects of H_2_O_2_. Whatever the exact mechanism, the increased sensitivity of tumor cells to killing by H_2_O_2 _may provide the specificity and "therapeutic window" for the antitumor therapy [11]. Colloidal silver is a common substance used by the Mexican people for disinfecting foods and water for their consumption, and at this time there is not a report on potential secondary effects related to this treatment; this also agreed with a recent study in mice performed in our laboratory, where colloidal silver was provided in the water at 10- and 50-fold higher concentrations than the recommended by the manufacturer during one year without finding any alterations in the evaluated parameters (fertility, birth, and tumors development) (data not shown). However, more studies are needed to elucidate the mechanism of colloidal silver action, with the aim of developing new strategies for the treatment of cancer and other illness, with lower cost and effectiveness. Therefore, it can be suggested that colloidal silver treatment may be used as an alternative treatment against cancer. However, the mechanism and pathways by which colloidal silver induced cytotoxic activity on MCF-7 human breast cancer cell line need further investigation.

## Conclusions

The overall results indicated that the colloidal silver has antitumor activity through induction of apoptosis in MCF-7 breast cancer cell line, suggesting that colloidal silver might be a potential alternative agent for human breast cancer therapy.

## List of abbreviations

PBMC: peripheral blood mononuclear cells; LDH: lactate dehydrogenase; NO: nitric oxide; Gpx: glutathione peroxidase; SOD: superoxide dismutase; CAT: catalase; ROI: reactive oxygen intermediates.

## Competing interests

The authors declare that they have no competing interests.

## Authors' contributions

MAFM conceived of the study, participated in its design and coordination, performed the statistical analysis and drafted the manuscript. EMG participated in drafting the manuscript. CASR carried out the proliferation, cell viability, apoptosis, and antioxidants assays, and drafted the manuscript. RAFG participated in drafting the manuscript. PZB participated in the design of the study and statistical analysis. PCT carried out Tunel Assay. JMAG participated in the draft preparation. DFMH participated in drafting the manuscript. RSTG and CRP participated in the design of study. All authors read and approved the final manuscript.

## References

[B1] Wadhera Akhil MD Fung Max Systemic argyria associated with ingestion of coloidal silver Dermatology 2005 11 1 15748553

[B2] Kim JS Kuk E Antimicrobial effects of silver nanoparticles Nanomedicine 2007 3 1 95 101 10.1016/j.nano.2006.12.001 17379174

[B3] Basu S Jana S Parde S Pal T Interaction of DNA bases with silver nanoparticles: assembly quantified throughout SPRS and SERS Colloid Interface 2008 321 2 288 93 10.1016/j.jcis.2008.02.015 18346751

[B4] Lansdown AB Silver in health care: antimicrobial effects and safety in use Dermatology 2006 33 17 34 10.1159/000093928 16766878

[B5] Asha Rani PV Prakash Hande M Suresh Valiyaveettil Anti-proliferative activity of silver nanoparticles BMC Cell Biology 2009 10 65 10.1186/1471-2121-10-65 PMC2759918 19761582

[B6] National Cancer Institute Breast Cancer Treatment 2007 http://www.cancer.gov

[B7] Gonzales Rengifo G Gonzales Castañeda C Rojas Tubeh Overexpression of genes of glycolytic pathway enzymes in cancer cells Acta Med. Peruana 2007 24 3 187 197

[B8] Mazurek S Zander U Eigenbrodt E *In vitro *effect of extracellular AMP on MCF-7 breast cancer cells: inhibition of glycolysis and cell proliferation Cell Physiol 1992 153 3 539 49 10.1002/jcp.1041530315 1447315

[B9] Narayanan Sriram Enhancement of antioxidant defense system by Epigallocatechin-3-gallate during bleomycin induced experimental pulmonary fibrosis Bio Pharm 2008 31 7 1306 1311 10.1248/bpb.31.1306 18591765

[B10] Kim DW Hong GH Lee HH Choi SH Chun BG Won CK Hwang IK Won MH Effect of colloidal silver against the cytotoxicity of hydrogen peroxide and naphthazarin on primary cultured cortical astrocytes Neuroscience 2007 117 3 387 400 10.1080/00207450600592016 17365122

[B11] Balz Frei Stephen Lawson Vitamin C and cancer revisited PNAS 2008 105 32 11037 11038 10.1073/pnas.0806433105 PMC2516245 18682554

